# Molecular Models of STAT5A Tetramers Complexed to DNA Predict Relative Genome-Wide Frequencies of the Spacing between the Two Dimer Binding Motifs of the Tetramer Binding Sites

**DOI:** 10.1371/journal.pone.0160339

**Published:** 2016-08-18

**Authors:** Bangalore K. Sathyanarayana, Peng Li, Jian-Xin Lin, Warren J. Leonard, Byungkook Lee

**Affiliations:** 1 Laboratory of Molecular Biology, Center for Cancer Research, National Cancer Institute, National Institutes of Health, Bethesda, Maryland, 20892–4264, United States of America; 2 Laboratory of Molecular Immunology and the Immunology Center, National Heart, Lung, and Blood Institute, National Institutes of Health, Bethesda, Maryland, 20892–1674, United States of America; Tsinghua University School of Life Sciences, CHINA

## Abstract

STAT proteins bind DNA as dimers and tetramers to control cellular development, differentiation, survival, and expansion. The tetramer binding sites are comprised of two dimer-binding sites repeated in tandem. The genome-wide distribution of the spacings between the dimer binding sites shows a distinctive, non-random pattern. Here, we report on estimating the feasibility of building possible molecular models of STAT5A tetramers bound to a DNA double helix with all possible spacings between the dimer binding sites. We found that the calculated feasibility estimates correlated well with the experimentally measured frequency of tetramer-binding sites. This suggests that the feasibility of forming the tetramer complex was a major factor in the evolution of this DNA sequence variation.

## Introduction

STAT (Signal Transducer and Activator of Transcription) proteins are activated by interferons, cytokines, and growth factors as a critical cytoplasmic to nuclear signaling mechanism [1]. Classically, STAT proteins were shown to be activated by tyrosine phosphorylation, allowing their dimerization and nuclear translocation [2]. Subsequently, N-terminal domain (NTD) mediated dimerization of dimers to form tetramers was also demonstrated [3]. We previously showed that STAT5 tetramerization is critical for normal cytokine responses and immune function [4]. Moreover, STAT5 tetramers, but not dimers, were reported to be associated with leukemia development in mice [5]. Tetramerization of STAT1 is also vital for normal immune function [6]. Many specific genes have been shown to be activated by STAT protein tetramers, including those encoding IL-2 receptor α [7], interferon-γ [8], α2-macroglobulin [9, 10], and serine protease inhibitor 2.1 [11]. Together, these studies underscore the critical role of STAT tetramerization in vivo.

A STAT5A dimer binds to DNA by recognizing a γ-interferon-activated sequence (GAS) motif, with the consensus sequence TTCNNNGAA [12], through the two DNA binding domains (DBDs) of the dimer. A tetramer can form when two dimers bind to tandemly linked GAS and GAS-like motifs and their NTDs interact by dimerization [8, 13]. The spacing between the two GAS motifs varies and the genome-wide frequency of tetramer binding sites with different spacings is not random, with the gap length between the GAS motifs of ~11–12 base pairs (bps) the most frequent and secondary peaks at gap lengths of ~6–7 and 16–17 bps [4]. Since a GAS motif is 9 bps long, the center-to-center distance (CTCD) is 9 bps greater than the gap length. Accordingly, these peaks occur at multiples of ~5 bps in terms of CTCDs, approximately corresponding to the number of base pairs in a half helical turn of DNA and suggesting that the observed frequency pattern may relate to the likelihood of forming tetramers at different dimer separations. To test this hypothesis, here we report building STAT5A tetramer models on DNA, computing relative feasibility measures for forming them with all possible spacings between the two GAS motifs, and comparing the computed feasibility measures with experimentally measured frequencies of dimer spacings. We find that there is a high correlation between the two quantities.

## Results

**Fig 1:**
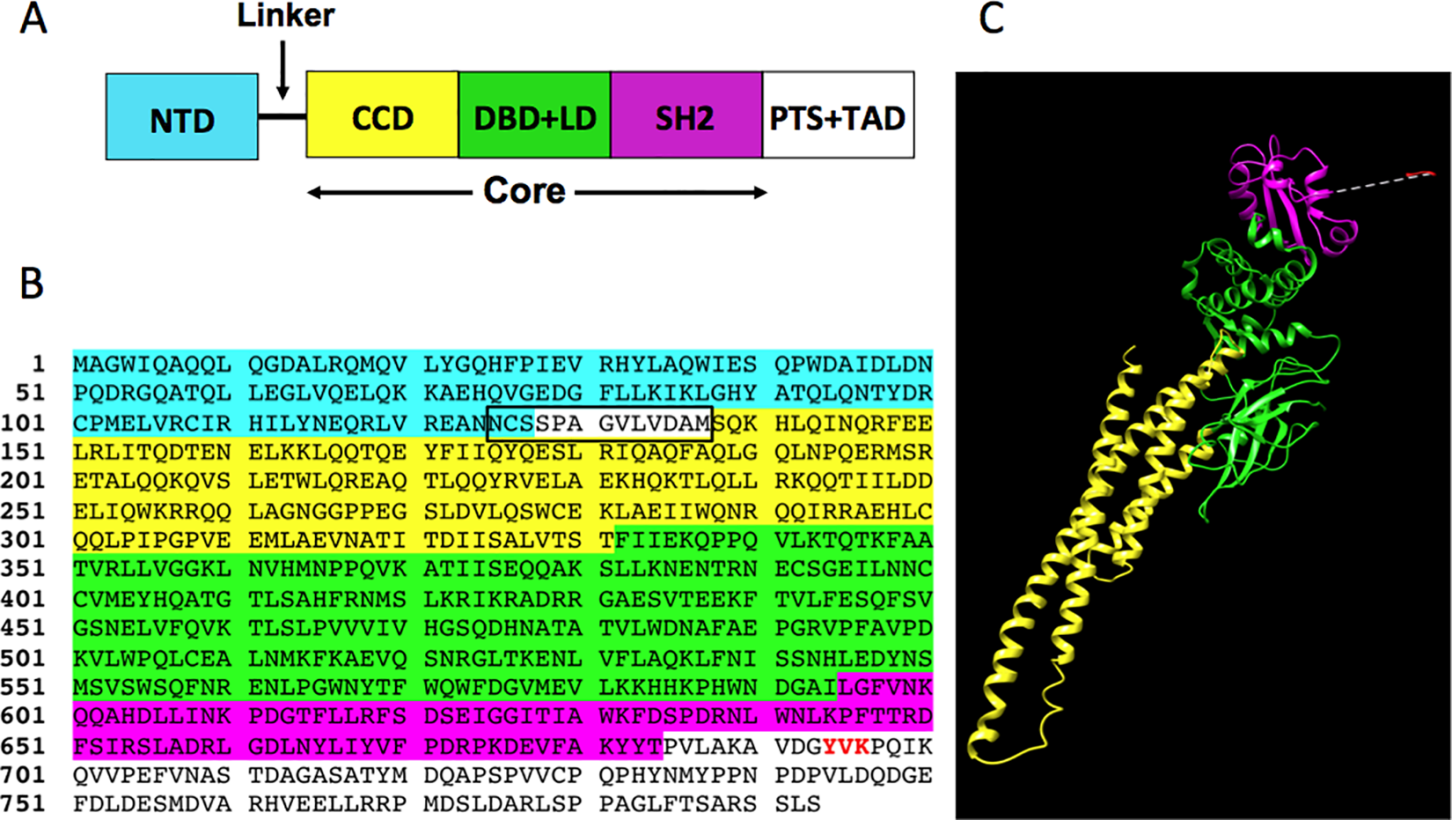
The domain structure of STAT proteins. **(A)** Names and positions of the six domains on the primary sequence: NTD (N-terminal domain), CCD (4-helix bundle coiled-coil domain), DBD (DNA-binding domain), LD (linker or connector domain), SH2, and TAD (transactivation domain). We treated the DBD and LD as one combined domain, as SCOP [17] does for STAT3β. There is a short flexible 13-residue linker between the NTD and CCD, which partly overlaps with the NTD. There is also a phospho-tyrosine containing segment (PTS) between the SH2 and TAD, which we combined with the TAD. See Methods for more detail. (**B)** The sequence of mouse STAT5A (NCBI Accn# CAA88419.1). The residues are highlighted according to the domains to which they belong, using the same coloring scheme used in (A). The 13-residue linker is boxed. The phospho-tyrosine residue (Y694) and the two following residues (V695 and K696) are shown in red. **(C)** STAT5A domains in the modeled structure of the STAT5A core, which includes the CCD, DBD, LD, SH2 and the residues 694–696 of the PTS. Dotted line represents the connecting residues (685–693) that were not included in the model.

**Fig 2:**
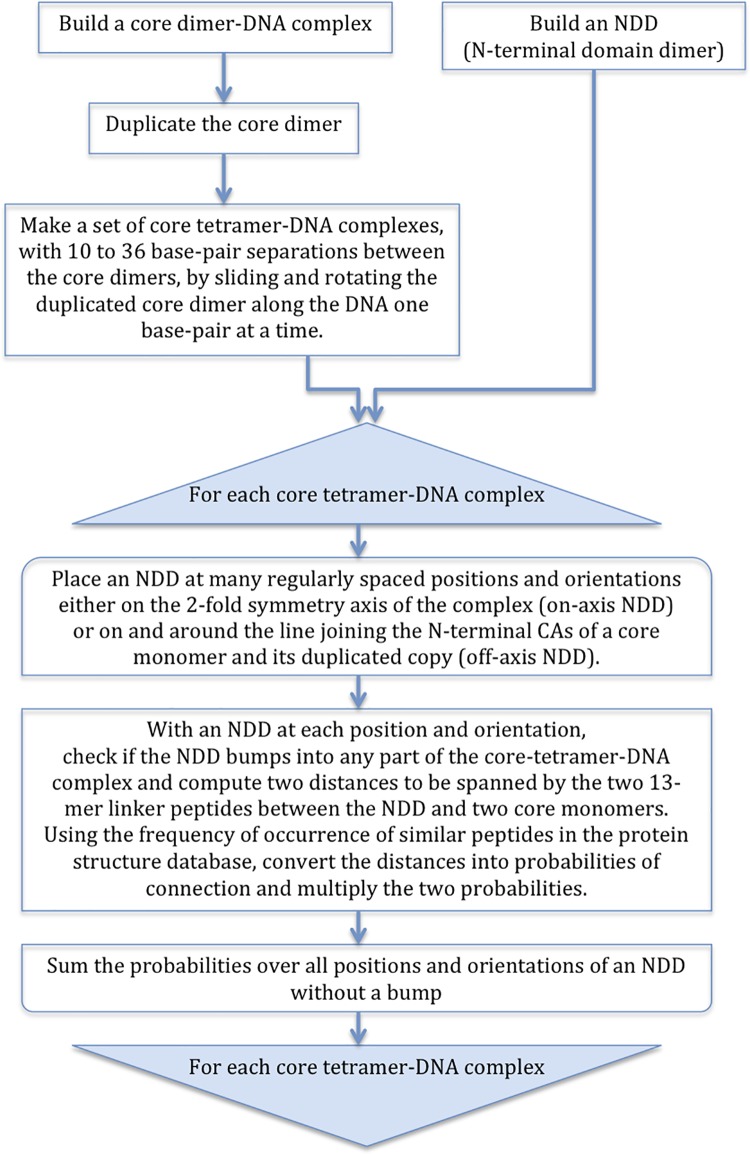
A flow chart of the model building procedure.

STAT proteins contain six domains [14–16] and connecting peptides (Fig 1). The “core” of the molecule is made of the four middle domains, lacking the N-terminal (NTD) and the transactivation (TAD) domains and all but three residues of the phospho-tyrosine containing segment (PTS). There is a 13-residue flexible linker between the NTD and the core. Tetramer models of the DNA-bound phosphorylated core at a particular DNA spacing were built before for STAT5A [12] and STAT1 [15]. We built tetramer models of STAT5A with all spacings between the dimer binding sites and included considerations of the N-terminal dimer formation. A flow chart of our model building process is given in Fig 2. We first built a DNA-bound, phosphorylated core dimer model using the crystal structures of STAT5A in its un-phosphorylated form (1y1u) [16] and of STAT3β in its phosphorylated dimer form interacting with DNA (1bg1) [14]. The core dimer was then duplicated and the duplicated dimer rotated and slid, one base pair at a time, along an ideal B-DNA with 10.5 bps per turn (see S1 Movie). The N-terminal domain dimer (NDD) that forms between two core dimers was built separately by homology modeling using the STAT4 NDD structure (1bgf) as a template. For each core dimer pair with particular DNA spacing, the NDD was then placed at many different locations and orientations and the feasibility was assessed as to whether the NDD could be connected to the two core dimers by means of the 13-residue linker on each side of the NDD.

**Fig 3:**
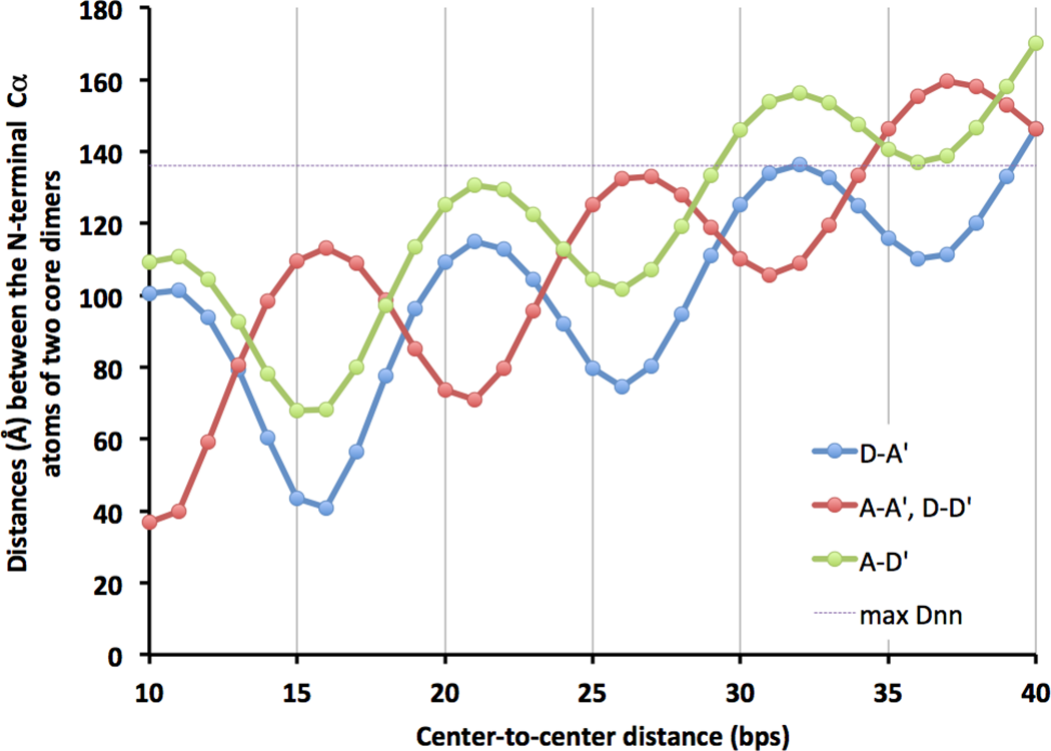
Distances between the N-terminal Cα atoms of two core monomers D-A’ (blue), A-D’ (green), and A-A’ or D-D’ (red) at the CTCD values indicated on the x-axis. The distance between the monomers D-D’ is the same as that between the monomers A-A’. The dotted line is at the distance of 136 Å, which is an expected upper limit of distances that can be spanned by an NDD with two 13-mer peptides. See Methods for the procedure used to calculate these distances.

We labeled the two monomers of one core dimer as A and D and the corresponding monomers in the duplicated dimer as A’ and D’. As the A’-D’ core dimer is slid along the DNA away from the A-D core dimer, it also rotates around the DNA axis and the distance between a pair of N-termini of moving and stationary dimers oscillates while it generally increases (Fig 3). Two core dimers that are 9 or less CTCDs apart had clashes between them. Also, two dimers could not be connected by an N-terminal domain dimer (NDD) when the CTCD was greater than 39 (Fig 3 and Methods). Therefore, we built models with CTCD values from 10 to 39.

**Fig 4:**
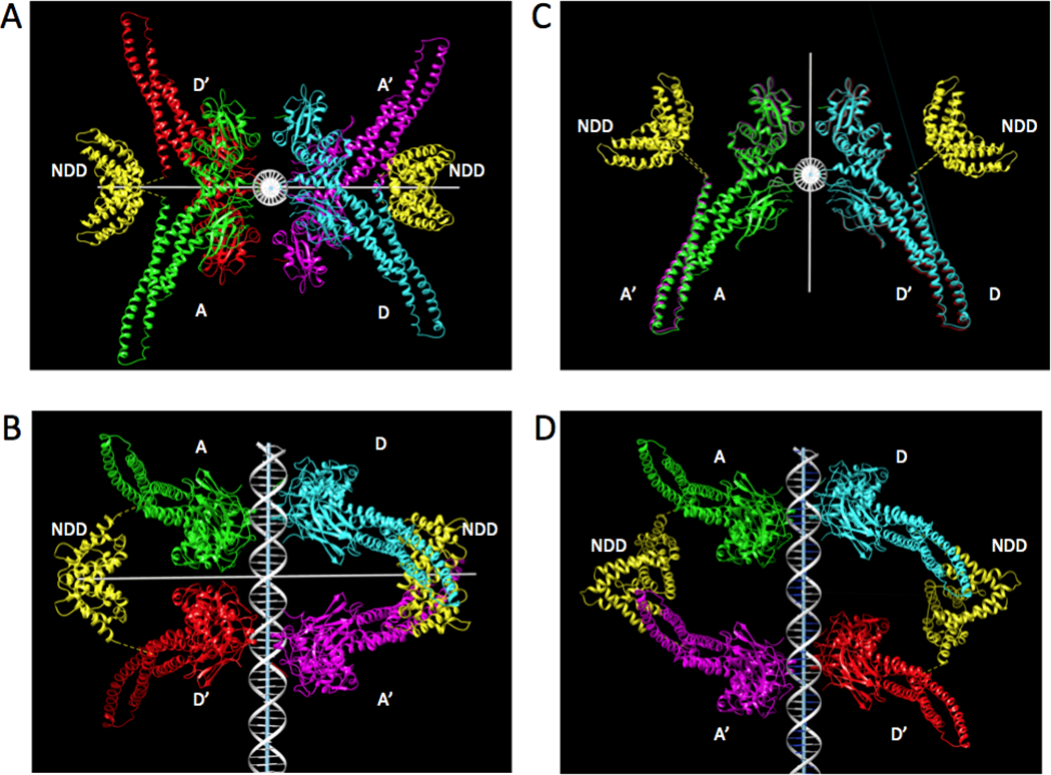
Two examples of the modeled tetramer structure. Left panels (NDD on-axis): The plan view (**A**) and elevation (**B**) of the tetramer model at the CTCD of 16; the horizontal white line is the 2-fold symmetry axis. Right panels (NDD off-axis): The plan view (**C**) and elevation (**D**) of the tetramer model at the CTCD of 21; the 2-fold axis is vertical in (C) and along the viewing direction in (D). In each panel, DNA is white; the core monomers are green (core A), blue (core D), magenta (A’), and red (D’); and NDDs are yellow. The linkers between the C-terminals of the NDD and the N-terminals of the core are indicated as dotted yellow lines.

There are two topologically distinct tetramers depending on which two monomers are connected by an NDD. All core tetramer models have a 2-fold symmetry axis, which runs perpendicular to the DNA axis and relates monomers A to D’ and D to A’ (Fig 4). An NDD can connect the symmetry-related monomers, which come near each other on the same side of DNA when the two core dimers are rotated approximately 180° from one another (‘staggered’ tetramer), at CTCD values of ~15 or 25 (green and blue lines in Fig 3; Fig 4A and 4B; and S2 Movie). Since an NDD is itself 2-fold symmetric, we placed these NDDs on the symmetry axis, one on each side of DNA (on-axis NDDs). Another type of tetramer results when an NDD connects monomers A and A’, in which case the symmetry-related NDD can connect D and D’. These monomers come near each other when the two core dimers are related essentially by translation along the DNA axis with no or relatively small rotation (‘eclipsed’ tetramer) at CTCD values of ~10, 20 or 30 (red line in Fig 3; Fig 4C and 4D; and S3 Movie). The NDDs in this case are away from the symmetry axis (off-axis NDDs).

For both types of tetramers, the separately built NDD was placed at many discrete points and orientations, and the probability of connecting each to a pair of core monomers was assessed (see Methods). The relative feasibility, *F*_*2*_, of forming a tetramer using one or two NDDs was calculated as the sum of these probabilities. We note that when only one NDD connects the two dimers, there are two other NTDs per tetramer, which are free and can be used to form a higher order oligomer; however, we do not consider higher oligomers in this paper. The relative feasibility, *F*_*4*_, of forming a tetramer using two NDDs, which may be required for the stable cooperative tetramer formation on DNA, is the product of the sums of the probabilities with NDDs on each side of DNA.

**Fig 5:**
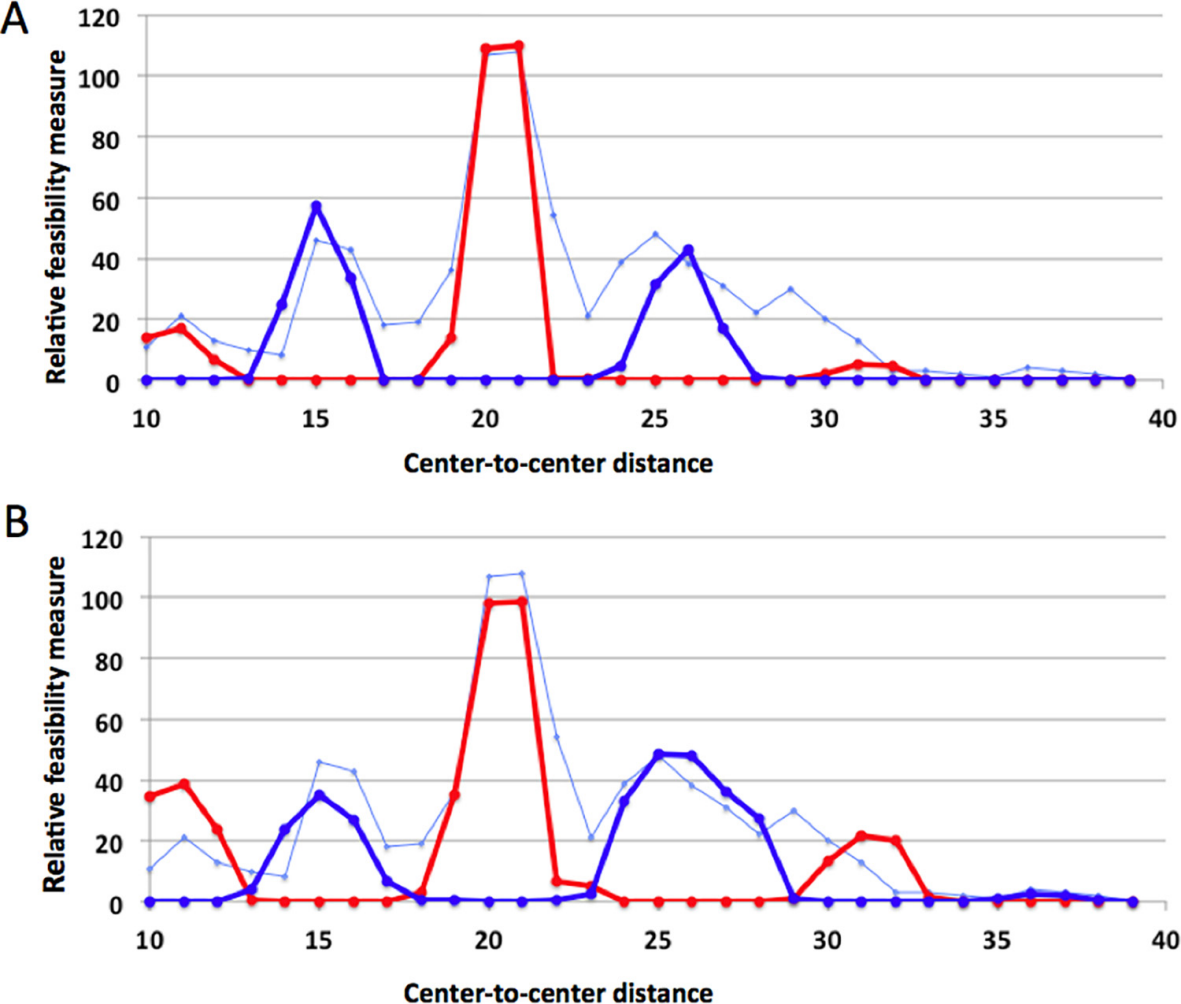
Computed scaled feasibility measures of the tetramer formation vs. CTCD using two NDDs (**A**) or at least one NDD (**B**). The red and blue points are for the ‘eclipsed’ and the ‘staggered’ tetramers, respectively, with the NDD off- and on-axis, respectively. The red and blue points were scaled so as to put them on the same scale as the experimental counts (shown in thin blue line) of tetramer binding sites in the mouse genome. For this figure, we used the DNA with 10.5 bps per helical turn. The results using the 10.0 bp DNA are given in Fig 6.

The computed feasibility measures are given in Table 1 and shown in Fig 5A (for *F*_*4*_) and Fig 5B (*F*_*2*_). Since NDDs at on-axis positions are constrained to lie on the symmetry axis whereas off-axis positions have no such constraint, there are many more possible positions for off-axis NDDs, which increases the feasibility values. Therefore, we scaled the on- and off-axis feasibility measures separately so that the sum best matched the experimental frequencies (see Methods). Both Fig 5A and 5B show five peaks, at CTCDs of 10–11, 15, 20–21, 25–26 and 31–32, similar to those seen in the plot of the experimentally measured frequencies (thin blue lines in Fig 5A and 5B). These peaks represent both ‘eclipsed’ (red peaks at CTCDs of around 10, 20, and 31) and ‘staggered’ (blue peaks at CTCDs of around 15 and 26) tetramers. The troughs are at or near the valleys in the experimental frequency distribution (e.g., at or near CTCD values of 13, 18, 23, and 28). The inter-dimer rotation angles in these tetramers are approximately ±90°. Note also that the computed feasibility measure is zero or close to zero for CTCD values larger than 32. This is close to the CTCD value of 31, beyond which the experimentally observed frequency of tetramer binding sites is also at the background level.

**Table 1:** Computed scaled feasibility measures using 10.5 bps/turn DNA.

CTCD	F2		F4	
	Off-axis	On-axis	Off-axis	On-axis
	307.43*	690.89*	4269.3*	89064.8*
**10**	**34.86**	**0.01**	**13.72**	**0.00**
**11**	**38.95**	**0.00**	**17.13**	**0.00**
**12**	**24.00**	**0.06**	**6.51**	**0.00**
**13**	**0.45**	**4.18**	**0.00**	**0.30**
**14**	**0.03**	**23.90**	**0.00**	**24.58**
**15**	**0.00**	**35.13**	**0.00**	**57.38**
**16**	**0.00**	**26.88**	**0.00**	**33.67**
**17**	**0.01**	**6.95**	**0.00**	**0.01**
**18**	**2.95**	**0.62**	**0.10**	**0.00**
**19**	**35.32**	**0.25**	**14.09**	**0.00**
**20**	**98.38**	**0.09**	**109.30**	**0.00**
**21**	**98.76**	**0.01**	**110.14**	**0.00**
**22**	**6.75**	**0.52**	**0.51**	**0.00**
**23**	**5.33**	**2.53**	**0.32**	**0.03**
**24**	**0.06**	**33.20**	**0.00**	**4.75**
**25**	**0.00**	**48.52**	**0.00**	**31.52**
**26**	**0.00**	**47.83**	**0.00**	**42.72**
**27**	**0.00**	**36.12**	**0.00**	**17.15**
**28**	**0.14**	**27.24**	**0.00**	**1.20**
**29**	**1.17**	**1.04**	**0.02**	**0.00**
**30**	**13.32**	**0.06**	**2.00**	**0.00**
**31**	**21.75**	**0.00**	**5.34**	**0.00**
**32**	**19.90**	**0.00**	**4.47**	**0.00**
**33**	**1.47**	**0.00**	**0.02**	**0.00**
**34**	**0.01**	**0.15**	**0.00**	**0.00**
**35**	**0.00**	**1.08**	**0.00**	**0.00**
**36**	**0.00**	**2.47**	**0.00**	**0.00**
**37**	**0.00**	**1.98**	**0.00**	**0.00**
**38**	**0.00**	**0.44**	**0.00**	**0.00**
**39**	**0.00**	**0.01**	**0.00**	**0.00**

* These are the scale factors used to put the computed values at the same scale as the experimental genomic frequencies.

There are some obvious differences. For example, the calculated values are zero or nearly zero at CTCD values around 13, 18, 23, and 29, where the experimental frequencies appear to be significant. Also, as noted above, there are clashes between the core dimers when the CTCD value is 9 (gap length 0), but the experimental data (Fig 4D of ref. [4]) show a small, non-zero frequency at this spacing. The main causes for these discrepancies are presumably the facts that our models are rigid and that the DNA maintains the ideal geometry. There are clashes between core dimers even at CTCDs of 10–12, but we ignored them because they are small in number (<10) and appear to be avoidable by local alterations of the conformation of flexible loops and/or by small bending and twisting of the DNA.

**Fig 6:**
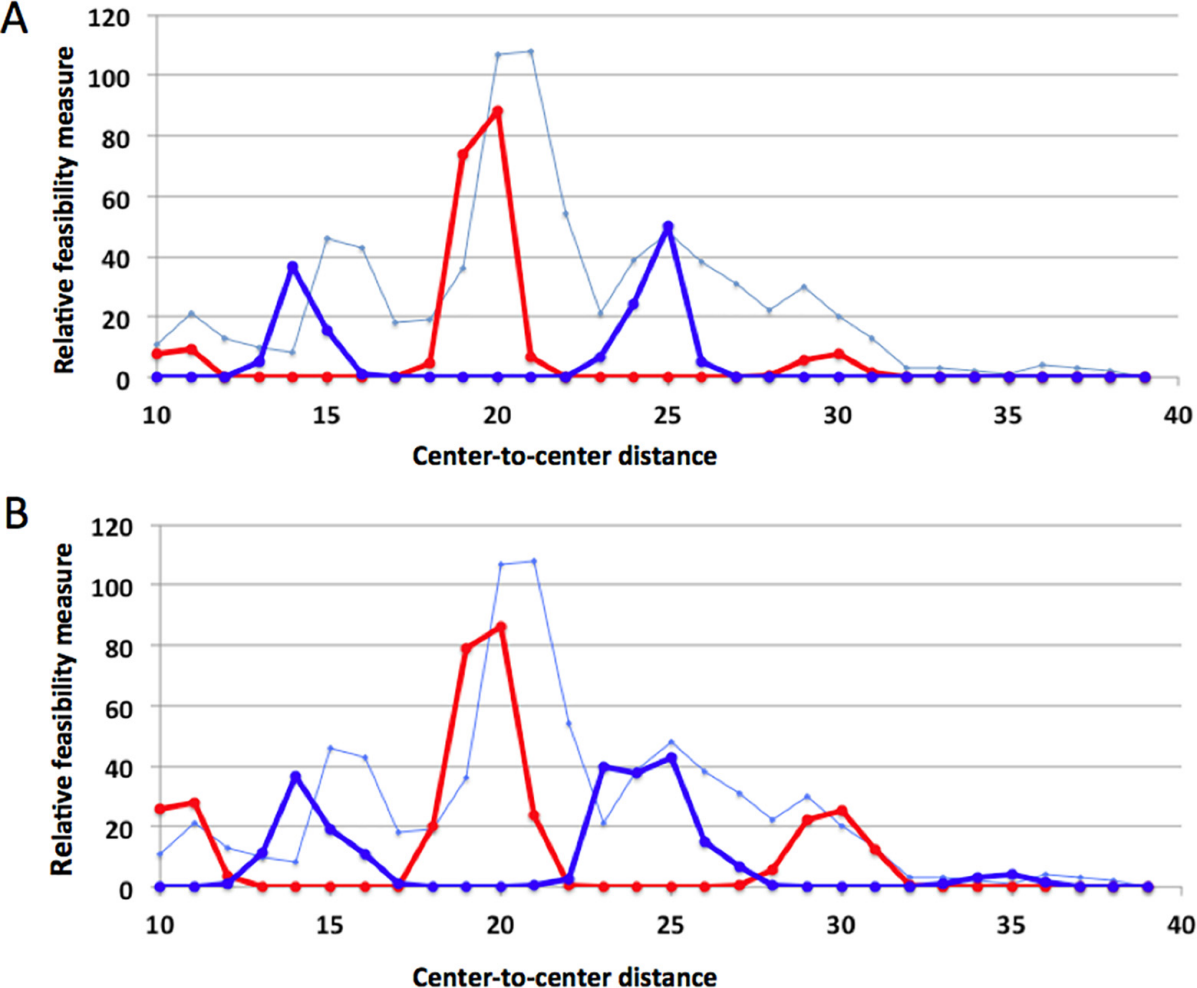
Computed scaled feasibility measures of the tetramer formation vs. CTCD using two NDDs (**A**) or at least one NDD (**B**) using the DNA with 10.0 bps per helical turn.

To see the effect of the twisting of DNA, we performed the same computations using a DNA with 10.0 bps per turn instead of 10.5 bps (Table 2). The calculated feasibility measures were similar for the two DNAs, but the peaks and troughs generally were shifted to the left with 10.0 bp DNA (Fig 6), so that the fit to the experimental frequency data was generally poorer than with 10.5 bp DNA (Fig 5). However, the position of the trough at a CTCD of 28 was nearly reproduced with 10.0 bp DNA. Thus, while the main features of the gap length dependence of the tetramer binding site frequencies can be understood without considering DNA and protein flexibility, such flexibility may have contributed to shape the finer features of the frequency distribution.

**Table 2:** Computed scaled feasibility measures using 10.0 bps/turn DNA.

CTCD	F2		F4	
	Off-axis	On-axis	Off-axis	On-axis
	235.58*	582.87*	2634.2*	59764.0*
**10**	**25.80**	**0.00**	**7.90**	**0.00**
**11**	**27.74**	**0.00**	**9.13**	**0.00**
**12**	**3.60**	**1.27**	**0.15**	**0.07**
**13**	**0.02**	**11.31**	**0.00**	**4.99**
**14**	**0.00**	**36.85**	**0.00**	**36.79**
**15**	**0.00**	**19.08**	**0.00**	**15.25**
**16**	**0.00**	**10.93**	**0.00**	**0.83**
**17**	**1.56**	**1.02**	**0.03**	**0.00**
**18**	**20.31**	**0.10**	**4.90**	**0.00**
**19**	**78.89**	**0.01**	**73.85**	**0.00**
**20**	**86.30**	**0.02**	**88.38**	**0.00**
**21**	**23.85**	**0.31**	**6.75**	**0.00**
**22**	**0.35**	**2.67**	**0.00**	**0.04**
**23**	**0.02**	**39.92**	**0.00**	**6.49**
**24**	**0.00**	**37.47**	**0.00**	**24.30**
**25**	**0.00**	**42.83**	**0.00**	**49.93**
**26**	**0.01**	**14.97**	**0.00**	**5.04**
**27**	**0.35**	**6.63**	**0.00**	**0.19**
**28**	**5.48**	**0.47**	**0.36**	**0.00**
**29**	**22.15**	**0.03**	**5.82**	**0.00**
**30**	**25.13**	**0.00**	**7.50**	**0.00**
**31**	**12.29**	**0.00**	**1.79**	**0.00**
**32**	**0.30**	**0.13**	**0.00**	**0.00**
**33**	**0.00**	**0.98**	**0.00**	**0.00**
**34**	**0.00**	**3.21**	**0.00**	**0.00**
**35**	**0.00**	**4.05**	**0.00**	**0.01**
**36**	**0.00**	**1.46**	**0.00**	**0.00**
**37**	**0.00**	**0.16**	**0.00**	**0.00**
**38**	**0.00**	**0.00**	**0.00**	**0.00**
**39**	**0.00**	**0.00**	**0.00**	**0.00**

* These are the scale factors used to put the computed values at the same scale as the experimental genomic frequencies.

## Discussion

Figs 5 and 6 clearly show that the gap length dependence of the calculated feasibility measures shares critical features with that of the experimentally measured frequency of tetramer binding sites in the mouse genome, including the number and position of the five peaks and four valleys. Our models and calculations, therefore, suggest that the peaks at or near the CTCD values of 10, 20 and 30 correspond to eclipsed tetramers connected by one or two off-axis NDDs while the peaks at or near CTCD values of 15 and 26 correspond to staggered tetramers connected by on-axis NDD(s).

In general, the frequency with which a particular spacing occurs in a genome is an evolutionary feature that needs not be related to the ease with which a tetramer can be formed. For example, the frequency of a spacing could be random or dictated by the biological usefulness of the target genes so long as a tetramer can be formed at that spacing. However, our study indicates that the frequency is in fact dictated mainly by the feasibility of forming a tetramer.

This point is most clear from the low but non-zero frequency of the spacing at which the calculated feasibility measure is zero or nearly zero, e.g. at the CTCD value of 18 or 22. The non-zero frequency of tetramer binding sites with these spacings indicates that a tetramer complex can be formed on DNA sites with one of these spacings, presumably because of the flexibility of DNA and of surface loops of the protein. On the other hand, the fact that the frequency is low at these spacings indicates that the frequency is not random but related to the feasibility of forming the tetramer complex.

We note here that our feasibility measure is essentially a weighted number of different positions and orientations of the N-terminal domain dimer (NDD) at which it can connect a pair of protein core dimers through the two 13-mer peptide linkers. It is not a full measure of the stability of the tetramer complex because it does not include the energy of protein-DNA interaction, calculation of which is computationally demanding. We note also that some of the binding sites with significant F2 values may involve higher order oligomerization, which we did not consider in this study.

On the basis of the observation that mutations on the surface of NDD affect the stability of the tetramer complex, Staab et al.[18] recently suggested a model of the tetramer complex in which the two core dimers are arranged in an eclipsed state and connected by one on-axis NDD sitting close to the DNA. Our calculations show that a model of this type is possible (although possible positions of NDD are not in the ‘front’ but on the ‘backside’ of DNA in between the SH2 domains, see Fig 4C and 4D) but is associated with a low feasibility measure. At the CTCD of 21, for example, when the two core dimers are maximally eclipsed for the 10.5 bps/turn DNA, the F2 value (Table 1) for the on-axis NDD is non-zero but small; in most positions, the NDD either clashes with some parts of the core or is too far from the N-termini of the core coiled coil domains for the 13-mer peptide linker to connect (see S1 Tables). It is possible that this model, with NDD at a few barely possible positions, is stabilized energetically by an interaction with DNA, which our calculations do not include (see above). It is interesting that the possibility of this model is not needed in order to produce a high feasibility at the CTCD of 21 because there are many off-axis NDD positions that can connect a pair of core dimers at this separation.

The reason that the number of tetramer binding sites in a genome should correlate with the feasibility of forming a tetramer is not entirely clear. We are not aware of any previous work reporting such a correlation between a feature of DNA sequence evolution and a measure of ease with which a protein-DNA complex can be formed. It is possible that there are many more weaker tetramer binding sites on the genome and that the observed frequency represents only those sites that have appreciable binding at the physiological concentration of STAT.

By estimating a measure of the feasibility of the tetramer formation, our model can therefore predict the number of such sites in the genome relative to those with other spacings. Given the high homology of human vs. mouse STAT5A as well as the homology of STAT5A to other STAT proteins, including particularly STAT5B but also STAT3, the models we have built here are applicable to other STAT proteins as well.

## Methods

### STAT5A sequence and domain definitions

The *Mus musculus* STAT5A protein sequence (NCBI Accn# CAA88419.1, http://www.ncbi.nlm.nih.gov/) used in this study and its domain boundaries are shown in Fig 1. The N-terminal domain (NTD, residues 1–127, highlighted cyan) corresponds to the visible residues in the STAT4 NTD structure [19]. The domain boundaries for the coiled-coil domain (CCD, 138–331, yellow), DNA binding domain and linker domain (DBD+LD, 332–594, green), and SH2 domain (595–684, magenta) correspond to the four domains of STAT3β structure according to the alignment between STAT3β and STAT5A[14]. The transactivation domain (TAD, 712–793, un-highlighted) and the linker between SH2 and TAD, which we call the phospho-tyrosine containing segment (PTS, 685–711, un-highlighted), also correspond to those defined for STAT3[14]. The domain boundaries are similar according to the STAT1 and STAT5A structures [15, 16], except that the SH2 domain in the STAT5A structure was defined to include the phospho-tyrosine containing segment.

Most of the residues of the PTS are not visible in the crystal structure of STAT5A [16] and the solution conformation of the few visible residues (residues 685–690) is uncertain because the corresponding residues are missing in the structure of STAT3β [14]. Presumably, this region of the sequence assumes flexible structures. It is poorly conserved among different STAT proteins except for three residues including the phosphorylated tyrosine residue 694 [14, 17, 20]. No crystal structure is available for the TAD, although NMR structures of this domain from STAT1 and STAT2 proteins in complex with TAZ1 and TAZ2 domains of CREB-binding protein (CBP) are available [21]. The TAD sequences are not similar among different STAT proteins including STAT5A [20] and are probably intrinsically unstructured in the absence of the cognate co-factors. We therefore excluded both PTS and TAD in our models except for residues 694–696 (YVK in red font in Fig 1B), which include the phospho-tyrosine and two additional residues.

### Construction of STAT5A core dimer models

Crystal structures for STAT5A in its un-phosphorylated form (1y1u) [16] and for STAT3β in its phosphorylated dimer form interacting with DNA (1bg1) [14] are available. Since STAT5A is homologous to STAT3β (32% identical by NCBI BLAST pairwise alignment), we built a model of phosphorylated, DNA bound STAT5A core dimer by using the STAT3β dimer-DNA structure (1bg1) and superimposing and separately replacing each of the core domains, CCD, DBD+LD, and SH2, by the corresponding domains in the structure of the un-phosphorylated STAT5A (1y1u). The resulting structure was not refined in any way, as we did not want to potentially deviate from the observed phosphorylated dimer-DNA complex structure. Residues 694–696 in the PTS of the STAT3β structure (1bg1) were retained in the core dimer model of STAT5A. The structure of the core dimer of STAT1 [15] is very similar to that of STAT3β, but we used STAT3β because of its greater sequence similarity to STAT5A.

### Construction of STAT5A core tetramer models

The 3D-DART webserver (http://haddock.chem.uu.nl/dna/dna.php) was used to build an ideal 60-mer B-DNA with 10.5 or 10.0 bps per turn. Two copies of the DNA-bound core STAT5A dimer, built as above, were placed on this DNA by superimposing the two 18-mer DNAs of the dimers on the 60-mer DNA with a desired base pair separation between them. The 18-mer DNAs were then removed.

### Construction of the N-terminal domain dimer (NDD)

The structure of the NDD is available for STAT4 (1bgf) [19]. Since the NTD of STAT5A is homologous to that of STAT4 (NCBI BLAST pairwise blast gives 34% identity), we built the NDD of STAT5A by homology modeling from the STAT4 NDD structure (1bgf) using swiss-pdb web server (http://swissmodel.expasy.org). The dimer interface used to build the dimer was the reinterpreted dimerization interface by Chen et al.[22].

### An upper limit of the distance between the core dimers

An upper limit of the distance between the core dimers is reached when the two core dimers are so far apart that their N-terminal domains (NTDs) cannot come close enough to dimerize. We determined this upper limit using the condition

Dnn−Dcc<2*Dmax

where *Dnn* is the distance between the N-terminal Cα atoms of the two CCDs that one potential NDD connects, *Dcc* is the distance between the Cα atoms of the two C-terminal residues (Asn 124) of the NDD, which is 55.8 Å in the model we built, and *Dmax* is the maximum possible end-to-end distance of the 13-mer peptide linker between NTD and CCD of a STAT5A monomer, which we took to be 40 Å (see below). Therefore, a condition for the upper limit is *Dnn* < 136 Å.

### Distance between two N-terminal Cα atoms of two dimers of a core tetramer

There are four possible core monomer pairs that may be linked by two N-terminal domains in-between: A-A’, A-D’, D-A’, and D-D’. Two of these (A-A’ and D-D’) are equivalent by 2-fold symmetry. Assuming that the DNA does not bend and the core dimers are rigid, the distances, *Dnn*, between the N-terminal Cα atoms of each of these pairs can be calculated using the following formulas:

$$Dnn=\sqrt{{\left(dz\right)}^{2}+{\left(dxy\right)}^{2}}$$

and

$$dz(AA^{\prime })=dz(DD^{\prime })=nz,$$

$$dz(AD^{\prime })=nz+dz(AD),$$

$$dz({DA}^{\prime })=nz-dz(AD),$$

$$dxy(AA^{\prime })=dxy(DD^{\prime })=2r\;sin\frac{n\alpha }{2},$$

$$dxy(AD^{\prime })=2r\;cos(\frac{n\alpha }{2}-\beta ),$$

$$dxy({DA}^{\prime })=2r\;cos(\frac{n\alpha }{2}+\beta ),$$

where letters in parenthesis after a distance indicate the core monomer pair, *n* is equal to the CTCD, *z* and *α* are the rise and the rotation angle per bp of the DNA double helix, and *dz(AD)*, *r*, and *β* are geometrical properties of a core dimer as described below. For the B-DNA with 10.5 bps per turn that we used, *z* = 3.37 Å and *α* = 34.3°.

Let the cylindrical coordinates of the N-terminal Cα atoms of the monomers A and D be *(z*, *r*, *φ)* with subscripts A and D respectively, with the z-axis along the DNA axis. Then *dz(AD) = zD–zA*, *r = rA = rD*, and *β = [180° - (φD- φA)]/2*. For the core dimer model we built, *dz(AD)* = 13.64 Å, *r* = 49.79 Å, and *β* = 0.4°.

### An upper limit of the spacing between the dimers

Fig 3 shows the calculated *Dnn* distances for CTCD values between 10 and 40. The A-A’ and D-D’ distances become larger than the upper limit of 136 Å, and therefore an eclipsed tetramer with an off-axis NDD will not form, when the CTCD value is larger than 34. The A-D’ distance is beyond the limit when the CTCD value is larger than 29 whereas the D-A’ distance reaches the limit at the CTCD value of 39. The experimental data show that the number of tetramer binding sites detected is very low when the CTCD value is larger than 31 (gap length of 22 in Fig 4D of ref. [4]), which suggests that tetramer formation with only one NDD connection is rare, although the little blips in frequency at CTCDs of 36 and 37 may be an indication that a tetramer with only one NDD, presumably on-axis connecting D and A’ monomers, can form.

### End-to-end distance distribution of the linker between the NTD and CCD

There are ten residues (residues 128–137: SPAGVLVDAM) between the N-terminal and coiled-coil domains that are not visible in the STAT5A crystal structure [16]. We assume that this peptide fragment is flexible. The fact that the corresponding part is missing in the full STAT1 structure (1yvl) is consistent with such flexibility. The residues flanking these linker residues on the N-terminal side are the C-terminal residues of the NTD, the last three of which appear to be flexible in the NTD structure of STAT4, being outside of the last helix. We included these three residues as a part of the linker, so that the length of the linker was increased to 13 residues (residues 125 to 137 shown boxed in Fig 1B: NCSSPAGVLVDAM). We correspondingly shortened the NTD by three residues at the C-terminal end; whenever we refer to the C-terminal end of the NTD or the NDD, we refer to this shortened end.

**Fig 7:**
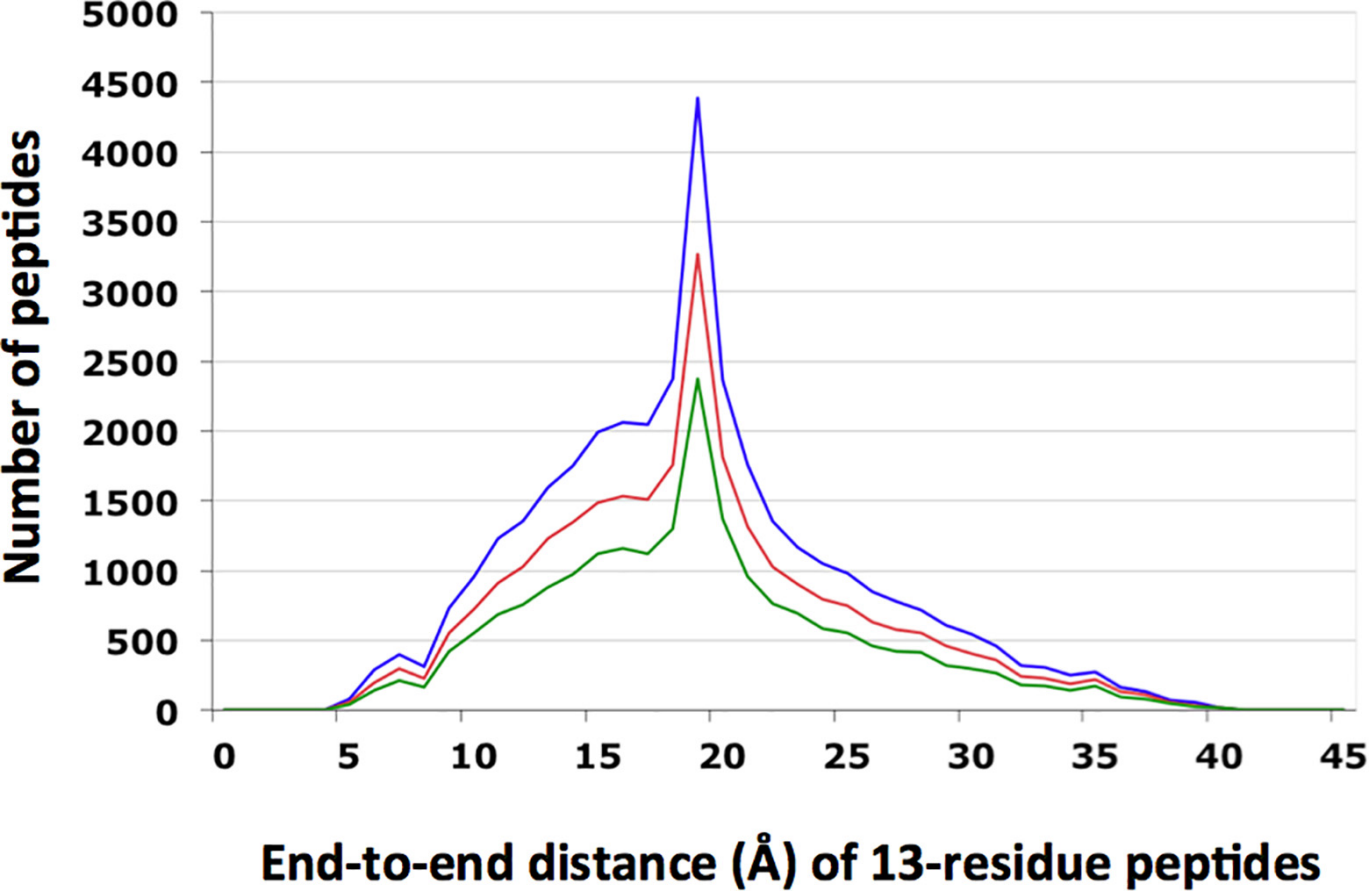
Histograms of the end-to-end distances of 13-residue peptides in protein structures in PDB, using only those peptides with the Blosum62 score greater than 8 (blue), 9 (red) or 10 (green) when compared to the linker sequence (shown in Box in Fig 1B) in STAT5A.

Instead of constructing possible structures for the 13-residue linker, we computed the end-to-end distance distribution of all 13-residue peptides in a subset of all the protein structures in the Protein Data Bank (PDB), which consisted of the structures with resolution < 2.5 Å, R-factor < 1.0 that were deposited before 03/23/2012 (19,412 chains in total). Each 13-residue peptide was aligned to the 13-residue linker sequence and the Blosum62 score [23] was computed for the alignment. The histograms of the end-to-end distances of the 13-residue peptides with high Blosum62 scores are shown in Fig 7. The end-to-end distances range between 4 and 40Å, with a peak at ~19Å. The overall distribution was rather insensitive to the particular Blosum62 score cutoff value. The histogram of the peptides with Blosum62 score greater than 8 was converted to the probabilities of the end-to-end distances in 1Å bins. These probabilities were used as the probability of linking the NTD to the CCD with the given end-to-end distance.

### Calculation of feasibility measures for tetramer formation with on-axis NDDs (Fig 4A and 4B)

An NDD was placed so that its 2-fold axis coincided with the 2-fold axis of the core tetramer-DNA complex. The NDD was then moved along the 2-fold axis on both sides of DNA at 10Å intervals. At each position, the NDD was flipped 180° around the vertical (perpendicular to the 2-fold) axis and both the flipped and un-flipped NDDs were rotated about the 2-fold axis at 30° intervals. At each position and orientation of an NDD, three numbers were calculated: the number of clashes between the NDD and the core tetramer and the two distances from the C-terminus of a monomer of the NDD to the N-termini of the two core monomers that are on the same side of DNA as the NDD. A “clash” is defined as an instance when a Cα to Cα non-bonded distance is less than 5Å or when a Cα atom of an NDD comes within van der Waals contact with a DNA atom, as defined by Chimera. The number of clashes and the two distances are reported in S1 Tables and S3 Tables for models with 10.5 and 10.0 bps/turn DNA, respectively. If a clash occurred, the probability *p(x*, *ϕ)* that a tetramer will form with an NDD at position *x* and orientation *ϕ* was set to zero. Otherwise, the probability was calculated as *p(x*, *ϕ)* = *p*_*1*_^*2*^ *+ p*_*2*_^*2*^, where *p*_*1*_ and *p*_*2*_ are the probabilities that a 13-mer peptide will have the end-to-end distance equal to the two distances calculated, respectively. Note that *p*_*1*_ and *p*_*2*_ denote probabilities of different ways of linking an NTD to the core dimer and that each needs to be squared because two symmetric connections are made in each case. These probabilities are also reported in the S1 Tables and S3 Tables.

We take the sum

$$F_{2}=\left[\sum_{x>0}p(x,\phi )\right]+\left[\sum_{x<0}p(x,\phi )\right]$$

as a relative measure of the feasibility of forming a tetramer linked by at least one on-axis NDD, which connects two core monomers. Here, the first summation is over all positions and orientations of an NDD on one side of DNA, connecting the core monomers A and D’, and the second summation is for positions of an NDD on the other side of DNA, connecting D and A’. We take the product

$$F_{4}=\left[\sum_{x>0}p(x,\phi )\right]∙\left[\sum_{x<0}p(x,\phi )\right]$$

as the relative feasibility measure for forming a tetramer with two connections involving both NDDs and all four core monomers. The computed values of the probability sums and of *F*_*2*_ and *F*_*4*_ are given in the “on-axis” columns of Table 3 for the 10.5 bps/turn DNA and of Table 4 for the 10.0 bps/turn DNA.

**Table 3:** Computed probability sums and raw feasibility measures using 10.5 bps/turn DNA.

CTCD	P*1000			Unscaled F2*1000		Unscaled F4*10000	
	Off-axis	On-axis		Off-axis	On-axis	Off-axis	On-axis
	P*	P_+_^#^	P_-_^&^				
**10**	**56.69**	**0.01**	**0.00**	**113.38**	**0.01**	**32.14**	**0.00**
**11**	**63.35**	**0.00**	**0.00**	**126.70**	**0.00**	**40.13**	**0.00**
**12**	**39.04**	**0.00**	**0.08**	**78.07**	**0.08**	**15.24**	**0.00**
**13**	**0.74**	**5.43**	**0.61**	**1.48**	**6.05**	**0.01**	**0.03**
**14**	**0.04**	**22.12**	**12.48**	**0.09**	**34.60**	**0.00**	**2.76**
**15**	**0.00**	**23.91**	**26.94**	**0.00**	**50.85**	**0.00**	**6.44**
**16**	**0.00**	**18.81**	**20.10**	**0.00**	**38.91**	**0.00**	**3.78**
**17**	**0.02**	**0.01**	**10.06**	**0.04**	**10.06**	**0.00**	**0.00**
**18**	**4.80**	**0.00**	**0.90**	**9.61**	**0.90**	**0.23**	**0.00**
**19**	**57.45**	**0.37**	**0.00**	**114.90**	**0.37**	**33.00**	**0.00**
**20**	**160.00**	**0.13**	**0.00**	**320.01**	**0.13**	**256.01**	**0.00**
**21**	**160.62**	**0.01**	**0.00**	**321.24**	**0.01**	**257.98**	**0.00**
**22**	**10.98**	**0.74**	**0.00**	**21.96**	**0.75**	**1.21**	**0.00**
**23**	**8.67**	**3.57**	**0.09**	**17.35**	**3.66**	**0.75**	**0.00**
**24**	**0.10**	**46.91**	**1.14**	**0.19**	**48.05**	**0.00**	**0.53**
**25**	**0.00**	**64.76**	**5.47**	**0.00**	**70.22**	**0.00**	**3.54**
**26**	**0.00**	**61.43**	**7.81**	**0.00**	**69.23**	**0.00**	**4.80**
**27**	**0.00**	**48.30**	**3.99**	**0.00**	**52.29**	**0.00**	**1.93**
**28**	**0.22**	**39.08**	**0.34**	**0.45**	**39.43**	**0.00**	**0.13**
**29**	**1.91**	**1.51**	**0.00**	**3.81**	**1.51**	**0.04**	**0.00**
**30**	**21.66**	**0.09**	**0.00**	**43.32**	**0.09**	**4.69**	**0.00**
**31**	**35.37**	**0.00**	**0.00**	**70.73**	**0.00**	**12.51**	**0.00**
**32**	**32.36**	**0.00**	**0.00**	**64.72**	**0.00**	**10.47**	**0.00**
**33**	**2.40**	**0.00**	**0.00**	**4.80**	**0.00**	**0.06**	**0.00**
**34**	**0.01**	**0.21**	**0.00**	**0.02**	**0.21**	**0.00**	**0.00**
**35**	**0.00**	**1.57**	**0.00**	**0.00**	**1.57**	**0.00**	**0.00**
**36**	**0.00**	**3.58**	**0.00**	**0.00**	**3.58**	**0.00**	**0.00**
**37**	**0.00**	**2.87**	**0.00**	**0.00**	**2.87**	**0.00**	**0.00**
**38**	**0.00**	**0.63**	**0.00**	**0.00**	**0.63**	**0.00**	**0.00**
**39**	**0.00**	**0.01**	**0.00**	**0.00**	**0.01**	**0.00**	**0.00**

* ∑*p*(*z*,*r*,*ϕ*,*θ*)

^#^ ∑_*x*>0_*p*(*x*,*ϕ*)

^&^ ∑_*x*<0_*p*(*x*,*ϕ*)

**Table 4:** Computed probability sums and raw feasibility measures using 10.0 bps/turn DNA.

CTCD	P*1000			Unscaled F2*1000		Unscaled F4*10000	
	Off-axis	On-axis		Off-axis	On-axis	Off-axis	On-axis
	P*	P_+_^#^	P_-_^&^				
**10**	**54.75**	**0.00**	**0.00**	**109.51**	**0.00**	**29.98**	**0.00**
**11**	**58.87**	**0.00**	**0.00**	**117.74**	**0.00**	**34.66**	**0.00**
**12**	**7.63**	**1.26**	**0.91**	**15.27**	**2.17**	**0.58**	**0.01**
**13**	**0.05**	**12.95**	**6.45**	**0.10**	**19.40**	**0.00**	**0.84**
**14**	**0.00**	**51.21**	**12.02**	**0.00**	**63.23**	**0.00**	**6.16**
**15**	**0.00**	**12.82**	**19.91**	**0.00**	**32.73**	**0.00**	**2.55**
**16**	**0.00**	**0.78**	**17.98**	**0.00**	**18.76**	**0.00**	**0.14**
**17**	**3.30**	**0.00**	**1.74**	**6.61**	**1.74**	**0.11**	**0.00**
**18**	**43.11**	**0.17**	**0.00**	**86.23**	**0.17**	**18.59**	**0.00**
**19**	**167.44**	**0.02**	**0.00**	**334.87**	**0.02**	**280.35**	**0.00**
**20**	**183.17**	**0.03**	**0.00**	**366.35**	**0.03**	**335.52**	**0.00**
**21**	**50.63**	**0.50**	**0.04**	**101.26**	**0.53**	**25.63**	**0.00**
**22**	**0.75**	**4.45**	**0.13**	**1.49**	**4.58**	**0.01**	**0.01**
**23**	**0.05**	**66.86**	**1.62**	**0.10**	**68.49**	**0.00**	**1.09**
**24**	**0.00**	**57.17**	**7.11**	**0.00**	**64.28**	**0.00**	**4.07**
**25**	**0.00**	**59.41**	**14.06**	**0.00**	**73.47**	**0.00**	**8.35**
**26**	**0.01**	**21.82**	**3.86**	**0.03**	**25.68**	**0.00**	**0.84**
**27**	**0.75**	**11.09**	**0.28**	**1.49**	**11.38**	**0.01**	**0.03**
**28**	**11.64**	**0.81**	**0.00**	**23.27**	**0.81**	**1.35**	**0.00**
**29**	**47.01**	**0.05**	**0.00**	**94.02**	**0.05**	**22.10**	**0.00**
**30**	**53.35**	**0.00**	**0.00**	**106.69**	**0.00**	**28.46**	**0.00**
**31**	**26.09**	**0.00**	**0.00**	**52.17**	**0.00**	**6.81**	**0.00**
**32**	**0.64**	**0.21**	**0.00**	**1.28**	**0.21**	**0.00**	**0.00**
**33**	**0.00**	**1.69**	**0.00**	**0.00**	**1.69**	**0.00**	**0.00**
**34**	**0.00**	**5.50**	**0.01**	**0.00**	**5.51**	**0.00**	**0.00**
**35**	**0.00**	**6.94**	**0.02**	**0.00**	**6.96**	**0.00**	**0.00**
**36**	**0.00**	**2.51**	**0.00**	**0.00**	**2.51**	**0.00**	**0.00**
**37**	**0.00**	**0.27**	**0.00**	**0.00**	**0.27**	**0.00**	**0.00**
**38**	**0.00**	**0.00**	**0.00**	**0.00**	**0.00**	**0.00**	**0.00**
**39**	**0.00**	**0.00**	**0.00**	**0.00**	**0.00**	**0.00**	**0.00**

* ∑*p*(*z*,*r*,*ϕ*,*θ*)

^#^ ∑_*x*>0_*p*(*x*,*ϕ*)

^&^ ∑_*x*<0_*p*(*x*,*ϕ*)

### Calculation of feasibility measures for tetramer formation with off-axis NDDs (Fig 4C and 4D)

A lattice of grid points was set up using a cylindrical coordinate system. The axis of the cylinder was chosen as the line connecting the N-terminal ends of the monomers D and D’. The grid points had the coordinates *(z*, *r*, *ϕ)* where *z*, *r*, and *ϕ* are the distance along, radial distance from, and the azimuthal angle around the cylinder axis, respectively. The distances *z* and *r* were varied in 10 Å intervals and *ϕ* was varied differently depending on the value of *r* in such a manner that the grid points were separated roughly by 10 Å in all directions. At each grid point, the NDD was placed with the NDD line, defined as the line joining the two C-termini of the NDD monomers, parallel to the *z*-axis and then rotated about the NDD line at an interval of 60°. At each position and orientation of the NDD, again three numbers were calculated: (1) the number of clashes between the NDD and the core tetramer, (2) the distance from the N-terminus of monomer D to the nearer of the two C-termini of the NDD, and (3) the distance between the N-terminus of D’ to the other C-terminus of the NDD. These data are reported in S2 Tables and S4 Tables for models with 10.5 and 10.0 bps/turn DNA, respectively. If a clash occurred, the probability *p(z*, *r*, *ϕ*, *θ)* that a tetramer will form with an NDD at position *(z*, *r*, *ϕ)* and orientation *θ* was set to zero. Otherwise, the probability was calculated for the position and orientation as *p(z*, *r*, *ϕ*, *θ)* = *p*_*1*_. *p*_*2*_, where *p*_*1*_ and *p*_*2*_ are the probabilities that a 13-mer peptide will have the end-to-end distance equal to the two distances calculated, respectively. These probabilities are also reported in the S2 Tables and S4 Tables.

We then take the sum form

$$F_{2}=2\sum p(z,r,\phi ,\theta )$$

as the measure of the feasibility of forming a tetramer with at least one off-axis NDD, which connects two core monomers. Here, the summation is over all positions and orientations of an NDD connecting monomers D and D’. The probability is doubled because for every D-D’ connection, a symmetry-related A-A’ connection is also possible. We take the product form

$$F_{4}={\left[\sum p(z,r,\phi ,\theta )\right]}^{2}$$

as the feasibility measure for forming a tetramer with two connections involving both NDDs. The computed values of the probability sum and of *F*_*2*_ and *F*_*4*_ are given in the “off-axis” columns of Table 3 for the 10.5 bp/turn DNA and of Table 4 for the 10.0 bp/turn DNA.

### Scaling on- and off-axis feasibility measures

The feasibility of forming a tetramer with a particular CTCD value is the weighted sum of those with NDD at the on- and off-axis positions. The weights were determined so as to minimize the difference between the weighted sum and the experimentally measured number of tetramer binding sites. Thus, we determined the weights *w*_*j*_, where *j* = 1 or 2 for the on- or off-axis feasibilities, respectively, which will minimize *S = Σ*_*I*_ *(c*_*i*_*−n*_*i*_*)*^*2*^, where *c*_*i*_ *= Σ*_*j*_ *w*_*j*_*F*_*ji*_ is the calculated feasibility measure for on- (*j* = 1) or off- (*j* = 2) axis position for the CTCD value *i*, and *n*_*i*_ is the experimentally measured number of tetramer binding sites at the CTCD value *i*. The solution of the minimization problem is: *w = a*^*-1*^*b*, *a*_*jk*_ *= Σ*_*i*_*F*_*ji*_*F*_*ki*_, and *b*_*j*_ *= Σ*_*i*_*F*_*ji*_*n*_*i*_. The computed *w* values are 690.9 and 307.4, respectively, for the on- and off-axis *F*_*2*_ and 89064.8 and 4269.3 for *F*_*4*_ using 10.5 bps/turn DNA. For the 10.0 bps/turn DNA, the corresponding numbers are 582.9 and 235.6 for *F*_*2*_ and 59764.0 and 2634.2 for *F*_*4*_. The residual root-mean-square deviation between the experimental frequency and the scaled *F*_*2*_ and *F4* values were 14.1 and 16.9, respectively, for the 10.5 bps/turn and 23.8 and 27.8 for the 10.0 bps/turn DNA.

## Supporting Information

S1 Movie The core tetramer models of STAT5A at CTCD values of 0 and 13–23. The movie starts with the model with CTCD = 0 showing the two dimers superimposed on one another, looking down along the DNA axis. Then it shows other models by rotating and translating the “back” dimer with respect to the stationary “front” dimer. The models with CTCD values 13, 16, 21, and 23 are rotated around the X-axis to produce a 3D feel.
(MOV)

S2 Movie The “staggered” full tetramer model at the CTCD of 16. Two views of this model are shown in Fig 4A and 4B. In this movie, the same model is shown rotated in all different directions to give a 3D feel.
(MOV)

S3 Movie The “eclipsed” full tetramer model at the CTCD of 21. Two views of this model are shown in Fig 4C and 4D. In this movie, the same model is shown rotated in all different directions to give a 3D feel.
(MOV)

S1 Tables Distance-overlap-probability tables for on-axis NDDs with 10.5 bps/turn DNA. This is a Microsoft Excel file, which contains 26 Excel sheets, one sheet for each CTCD in the range of 10 and 36. Each sheet gives data on the tetramer model with the 10.5 bps/turn DNA and the N-terminal domain dimer (NDD) positioned on the tetramer 2-fold axis. Each sheet is made of 17 tables. **Table A** occupies columns A-P. **Column A**: The *x*-coordinate (position along the tetramer 2-fold axis) of the NDD (in Å). Position zero is on the DNA axis. **Column B**: The rotation angle *ϕ* (in degrees) of the NDD around the 2-fold axis. **Column C**: Number of clashes with the NDD unflipped. **Column D**: Number of clashes with the NDD flipped (rotated by 180°) around the vertical axis (parallel to the DNA axis). **Columns E, F, G and H**: Distance (in Å) from one of the two C-terminal CAs of the NDD to the N-terminal CA of the core monomers A’, A, D’ and D, respectively. **Columns I to L**: Same as above except from the other C-terminal CA of the NDD. **Column M**: Average of distances in columns E and L, which are equivalent by the 2-fold symmetry. **Column N**: Average of distances in columns F and K, which are equivalent by the 2-fold symmetry. **Column O**: Average of distances in columns G and J, which are equivalent by the 2-fold symmetry. **Column P**: Average of distances in columns H and I, which are equivalent by the 2-fold symmetry. **Table B (D1 = 1-A’/2-D)** occupies columns R-X and rows 1–19. **Table C (D2 = 1-A/2-D’)** occupies columns Z-AF and rows 1–19. **Table D (D3 = 1-D’/2-A)** occupies columns R-X and rows 22–40. **Table E (D4 = 1-D/2-A’)** occupies columns Z-AF and rows 22–40. Above four tables are columns M, N, O, and P, respectively, re-written in a 2-dimensional form, with rows for angles and columns for distances. **Table F** occupies columns AH-AN and rows 1–19. **Table G** occupies columns AH-AN and rows 22–40. These are columns C and D, respectively, re-written in a 2-dimensional form, with rows for angles and columns for distances. **Table H (A’-D)** occupies columns R-X and rows 44–62. **Table I (A-D’)** occupies columns Z-AF and rows 44–62. **Table J (D’-A)** occupies columns R-X and rows 66–83. **Table K (D-A’)** occupies columns Z-AF and rows 66–83. These are squared probabilities of a 13-mer peptide to have distances given in corresponding tables B-E, according to the data of Fig 7 or its digital form given in columns O-Q in any of the off-axis tables given in S2 Tables. **Table L** occupies columns AH-AN and rows 44–62. **Table M** occupies columns AH-AN and rows 66–83. An entry in these tables is 1 or 0 if the corresponding entry in tables F and G is 0 or non-zero, respectively. **Tables N, O, P, and Q** give *p(x*, *ϕ)* values described in the main text. **Table N (A’-D, U)** occupies columns R-X and rows 86–104. This is the sum of tables H and K, multiplied by table L. **Table O (A-D’, U)** occupies columns Z-AF and rows 86–104. This is the sum of tables I and J, multiplied by table L. **Table P (A’-D, F)** occupies columns R-X and rows 107–125. This is the sum of tables H and K, multiplied by table M. **Table Q (A-D’, F)** occupies columns Z-AF and rows 107–125. This is the sum of tables I and J, multiplied by table M. **The last rows in columns S and T** give the sum of all entries of the tables N and P (column S) and of the tables O and Q (column T). These are the probabilities (sum of *p(x*, *ϕ)*) that an on-axis NDD will connect a symmetry-related pair of monomers, either A’ and D (column S) or A and D’ (column T).
(XLSX)

S2 Tables Distance-overlap-probability tables for off-axis NDDs with 10.5 bps/turn DNA. This is a Microsoft Excel file, which contains 26 Excel sheets, one sheet for each CTCD in the range of 10 and 36. Each sheet gives two tables. **Table A** occupies columns A to K. It gives data on the tetramer model with the 10.5 bps/turn DNA and the N-terminal domain dimer (NDD) positioned off the tetramer 2-fold axis. Each line gives a particular position and orientation of the NDD and the distance and probability of connection of the NDD to the two core monomers D and D’. The position is given in cylindrical coordinate system, of which the z-axis coincides with the line connecting the N-terminal ends of D and D’ and the origin of the coordinate system is at the halfway point of the two N-termini. **Column A**: Radial *r*-coordinate (in Å) of the center of the NDD. **Column B**: Azimuthal angle (in degrees) of the center of the NDD. (Ignore the column heading ‘theta’–this is the *ϕ* angle in the main text.) **Column C**: Axial *z*-coordinate (in Å) of the center of the NDD. **Column D**: Rotation angle (in degrees) of the NDD around its own axis. (Ignore the column heading ‘phi’–this is the *θ* angle in the main text.) **Column E**: Distance (in Å) from the N-terminal CA of the core monomer D to the nearest of the two C-terminal CAs of the NDD. **Column F**: Distance (in Å) from the N-terminal CA of the core monomer D’ to the other C-terminal CA of the NDD. **Column G**: Number of clashes. An entry of -1 indicates that at least one of the distances in columns E and F is outside of the range of 4–40 Å. **Columns H and I**: Probabilities given in Table B corresponding to the distances dist1 and dist2, respectively. **Column J**: Column G converted to 1 when there is no clash or 0 otherwise. **Column K**: Product of columns H, I and J. These are the *p(z*, *r*, *ϕ*, *θ)* values described in the main text. **The last row in column K** gives the sum of all entries of column K. This is the probability (sum of *p(z*, *r*, *ϕ*, *θ)*) that an off-axis NDD will connect a pair of non-symmetry related monomers. **Table B** occupies columns O to Q. This table gives, in digital form, the same data given in Fig 7 for BLOSUM62 score of 8 or greater (blue curve in Fig 7). The same table is given on each sheet of the Excel file for the off-axis NDD. **Column O**: End-to-end distance of the peptide, in Å truncated to an integer. **Column P**: Number of 13-mer peptides in PDB, with the BLOSUM62 score of 8 or greater when compared to the sequence of the 13-mer linker peptide in STAT5A, with the end-to-end distance in the given bin. **Column Q**: Normalized frequency.
(XLSX)

S3 Tables Distance-overlap-probability tables for on-axis NDDs with 10.0 bps/turn DNA. Same as S1 Tables except that the DNA has 10.0 bps/turn.
(XLSX)

S4 Tables Distance-overlap-probability tables for off-axis NDDs with 10.0 bps/turn DNA. Same as S2 Tables except that the DNA has 10.0 bps/turn.
(XLSX)

## References

[1] Leonard WJ, O'Shea JJ. Jaks and STATs: biological implications. Annu Rev Immunol. 1998;16:293–322. Epub 1998/05/23. 10.1146/annurev.immunol.16.1.293. . 9597132 10.1146/annurev.immunol.16.1.293

[2] Stark GR, Darnell JE Jr. The JAK-STAT pathway at twenty. Immunity. 2012;36(4):503–14. Epub 2012/04/24. 10.1016/j.immuni.2012.03.013. 10.1016/j.immuni.2012.03.013 22520844 PMC3909993

[3] Timofeeva OA, Tarasova NI. Alternative ways of modulating JAK-STAT pathway: Looking beyond phosphorylation. JAKSTAT. 2012;1(4):274–84. Epub 2013/09/24. 10.4161/jkst.22313 2012JAKS0059R [pii]. 10.4161/jkst.22313 24058784 PMC3670285

[4] Lin JX, Li P, Liu D, Jin HT, He J, Ata Ur Rasheed M, et al Critical Role of STAT5 transcription factor tetramerization for cytokine responses and normal immune function. Immunity. 2012;36(4):586–99. 10.1016/j.immuni.2012.02.017 22520852 PMC3551341

[5] Moriggl R, Sexl V, Kenner L, Duntsch C, Stangl K, Gingras S, et al Stat5 tetramer formation is associated with leukemogenesis. Cancer cell. 2005;7(1):87–99. . 15652752 10.1016/j.ccr.2004.12.010

[6] Begitt A, Droescher M, Meyer T, Schmid CD, Baker M, Antunes F, et al STAT1-cooperative DNA binding distinguishes type 1 from type 2 interferon signaling. Nat Immunol. 2014;15(2):168–76. Epub 2014/01/15. 10.1038/ni.2794. 10.1038/ni.2794 24413774

[7] John S, Vinkemeier U, Soldaini E, Darnell JE, Jr., Leonard WJ. The significance of tetramerization in promoter recruitment by Stat5. Mol Cell Biol. 1999;19(3):1910–8. 10022878 10.1128/mcb.19.3.1910 PMC83984

[8] Xu X, Sun YL, Hoey T. Cooperative DNA binding and sequence-selective recognition conferred by the STAT amino-terminal domain. Science. 1996;273(5276):794–7. Epub 1996/08/09. . 8670419 10.1126/science.273.5276.794

[9] Dajee M, Kazansky AV, Raught B, Hocke GM, Fey GH, Richards JS. Prolactin induction of the alpha 2-Macroglobulin gene in rat ovarian granulosa cells: stat 5 activation and binding to the interleukin-6 response element. Mol Endocrinol. 1996;10(2):171–84. Epub 1996/02/01. 10.1210/mend.10.2.8825557. . 8825557 10.1210/mend.10.2.8825557

[10] Zhang X, Darnell JE Jr. Functional importance of Stat3 tetramerization in activation of the alpha 2-macroglobulin gene. The Journal of biological chemistry. 2001;276(36):33576–81. Epub 2001/07/05. 10.1074/jbc.M104978200 M104978200 [pii]. . 11438543 10.1074/jbc.M104978200

[11] Bergad PL, Shih HM, Towle HC, Schwarzenberg SJ, Berry SA. Growth hormone induction of hepatic serine protease inhibitor 2.1 transcription is mediated by a Stat5-related factor binding synergistically to two gamma-activated sites. The Journal of biological chemistry. 1995;270(42):24903–10. Epub 1995/10/20. . 7559615 10.1074/jbc.270.42.24903

[12] Soldaini E, John S, Moro S, Bollenbacher J, Schindler U, Leonard WJ. DNA binding site selection of dimeric and tetrameric Stat5 proteins reveals a large repertoire of divergent tetrameric Stat5a binding sites. Mol Cell Biol. 2000;20(1):389–401. Epub 1999/12/14. 10594041 10.1128/mcb.20.1.389-401.2000 PMC85094

[13] Vinkemeier U, Cohen SL, Moarefi I, Chait BT, Kuriyan J, Darnell JE Jr. DNA binding of in vitro activated Stat1 alpha, Stat1 beta and truncated Stat1: interaction between NH2-terminal domains stabilizes binding of two dimers to tandem DNA sites. Embo J. 1996;15(20):5616–26. Epub 1996/10/15. 8896455 PMC452306

[14] Becker S, Groner B, Muller CW. Three-dimensional structure of the Stat3beta homodimer bound to DNA. Nature. 1998;394(6689):145–51. Epub 1998/07/22. 10.1038/28101. . 9671298 10.1038/28101

[15] Chen X, Vinkemeier U, Zhao Y, Jeruzalmi D, Darnell JE Jr., Kuriyan J. Crystal structure of a tyrosine phosphorylated STAT-1 dimer bound to DNA. Cell. 1998;93(5):827–39. Epub 1998/06/18. S0092-8674(00)81443-9 [pii]. . 9630226 10.1016/s0092-8674(00)81443-9

[16] Neculai D, Neculai AM, Verrier S, Straub K, Klumpp K, Pfitzner E, et al Structure of the unphosphorylated STAT5a dimer. The Journal of biological chemistry. 2005;280(49):40782–7. . 16192273 10.1074/jbc.M507682200

[17] Andreeva A, Howorth D, Chandonia JM, Brenner SE, Hubbard TJ, Chothia C, et al Data growth and its impact on the SCOP database: new developments. Nucleic acids research. 2008;36(Database issue):D419–25. Epub 2007/11/15. 10.1093/nar/gkm993. 18000004 10.1093/nar/gkm993 PMC2238974

[18] Staab J, Riebeling T, Koch V, Herrmann-Lingen C, Meyer T. The two interfaces of the STAT1 N-terminus exhibit opposite functions in IFNgamma-regulated gene expression. Mol Immunol. 2015;67(2 Pt B):596–606. 10.1016/j.molimm.2015.07.015. 10.1016/j.molimm.2015.07.015 26275341

[19] Vinkemeier U, Moarefi I, Darnell JE Jr., Kuriyan J. Structure of the amino-terminal protein interaction domain of STAT-4. Science. 1998;279(5353):1048–52. Epub 1998/03/07. . 9461439 10.1126/science.279.5353.1048

[20] Shen Y, Darnell JE Jr. Antiviral response in cells containing Stat1 with heterologous transactivation domains. Journal of virology. 2001;75(6):2627–33. . 11222685 10.1128/JVI.75.6.2627-2633.2001 PMC115886

[21] Wojciak JM, Martinez-Yamout MA, Dyson HJ, Wright PE. Structural basis for recruitment of CBP/p300 coactivators by STAT1 and STAT2 transactivation domains. Embo J. 2009;28(7):948–58. 10.1038/emboj.2009.30 19214187 PMC2670858

[22] Chen X, Bhandari R, Vinkemeier U, Van Den Akker F, Darnell JE Jr., Kuriyan J. A reinterpretation of the dimerization interface of the N-terminal domains of STATs. Protein Sci. 2003;12(2):361–5. Epub 2003/01/23. 10.1110/ps.0218903. 12538899 10.1110/ps.0218903 PMC2312425

[23] Henikoff S, Henikoff JG. Amino acid substitution matrices from protein blocks. Proc Natl Acad Sci U S A. 1992;89(22):10915–9. . 1438297 10.1073/pnas.89.22.10915 PMC50453

[24] Pettersen EF, Goddard TD, Huang CC, Couch GS, Greenblatt DM, Meng EC, et al UCSF Chimera—a visualization system for exploratory research and analysis. J Comput Chem. 2004;25(13):1605–12. Epub 2004/07/21. 10.1002/jcc.20084. . 15264254 10.1002/jcc.20084

